# Eliciting medication preferences of patients with type 2 diabetes under different insurance coverages in China

**DOI:** 10.3389/fpubh.2024.1413642

**Published:** 2024-10-25

**Authors:** Lvyun Zheng, Shimeng Liu, Zhigang Liu, Chenchen Cao, Wenjing Xue, Yingyao Chen, Jing Liu

**Affiliations:** ^1^School of Management, Hainan Medical University, Haikou, China; ^2^School of Public Health, Fudan University, Shanghai, China; ^3^NHC Key Laboratory of Health Technology Assessment (Fudan University), Shanghai, China

**Keywords:** insurance coverage, type 2 diabetes mellitus, medication preference, discrete choice experiment, China

## Abstract

**Objective:**

To understand the medication preference of type 2 diabetes mellitus (T2DM) patients with different insurance coverages, and to provide reference for improving the patient-centered clinical treatment decision.

**Methods:**

This study used Discrete Choice Experiment (DCE) to elicit preferences of T2DM patients with different insurance coverages in China. A multistage stratified cluster-sampling procedure for data collection and a total of 1,409 valid respondent were conducted.

**Results:**

Seven attributes have significant influence on the preference of T2DM patients with Urban Employee Basic Medical Insurance (UEBMI) and Urban and Rural Residents Basic Medical Insurance (URRBMI) (*p* < 0.05). T2DM patients with UEBMI pay the most attention to Gastrointestinal adverse events, while T2DM patients with URRBMI pay the most attention to the Treatment efficacy/reduction in HbA1c. Patients with different medical insurance have different willingness to pay for Cardiovascular benefits, Mode of administration and Weight change. When Gastrointestinal adverse events is changed from higher (40%) to none (0%), patients with UEBMI are willing to pay ¥523.49 more per month, while patients with URRBMI are only willing to pay ¥266.62; When the Treatment efficacy/reduction in HbA1c changes from poor (0.5%) to Highest (2.5%), patients with UEBMI are willing to pay ¥518.44 more per month, while patients with URRBMI are willing to pay ¥328.33 more per month. The Gastrointestinal adverse events and the Treatment efficacy/reduction in HbA1c are the primary factors for T2DM patients with UEBMI and URRBMI, followed by the Hypoglycemic risk.

**Conclusion:**

Physicians should consider patients’ medication preferences in clinical medication treatment of T2DM patients with different insurance coverages, make targeted treatment decisions, and improve patients’ medication compliance to achieve better treatment results.

## Introduction

The World Health Organization (WHO) Global Report on Diabetes shows that the number of adults living with diabetes has quadrupled since 1980, reaching 422 million, and is projected to rise to 693 million by 2045 (1, 2). According to the 10th edition of the Global Diabetes Map, China alone accounts for 141 million diabetes patients, primarily with type 2 diabetes mellitus (T2DM) (3). In China, T2DM patients can access medical insurance benefits for outpatient and inpatient care. The two main types of insurance—Urban Employee Basic Medical Insurance (UEBMI) and Urban and Rural Residents Basic Medical Insurance (URRBMI)—cover different populations and offer varying levels of medication reimbursement (4, 5). UEBMI mainly insures employed individuals and retirees, while URRBMI covers unemployed residents in urban and rural areas. These differences in insurance policies affect patients’ medication choices and preferences (6).

With the increasing prevalence of diabetes in China, the associated health complications and economic burdens have intensified, highlighting the need for effective disease management (7–9). Current guidelines emphasize patient-centered care, recommending that treatment decisions, particularly regarding second-line hypoglycemic drugs, should consider both clinical characteristics and patient preferences (10, 11). However, despite the growing focus on personalized treatment, there is limited evidence on how different insurance schemes influence patients’ drug preferences. The importance of understanding medication preferences, as these preferences can significantly influence medication adherence, treatment outcomes, and overall healthcare costs. By aligning treatment options with patient preferences, clinicians can improve patient engagement, optimize therapeutic outcomes, and potentially reduce the economic burden on the healthcare system.

Research suggests that patients’ drug preferences are shaped by various factors, including efficacy, safety, convenience, and cost (12). For example, high-income patients often prefer medications with stronger blood glucose control, while others may prioritize convenience or cost (12, 13). However, inadequate patient involvement in treatment decision-making can lead to poor medication adherence and suboptimal blood sugar control, thereby increasing healthcare costs (14–16). Understanding how insurance coverage impacts patients’ preferences is crucial for improving glycemic control and reducing economic burdens (17).

This study aims to address this gap by examining the drug choice preferences of T2DM patients under UEBMI and URRBMI. Using a Discrete Choice Experiment (DCE), we will measure how different insurance types influence patient preferences, with the goal of enhancing patient involvement, improving clinical outcomes, and promoting better medication adherence through shared decision-making (SDM).

## Materials and methods

This study use a multistage stratified cluster-sampling procedure that considers geographical region and economic development status for data collection. Firstly, Hainan and Shanxi province were selected based on their geographical location and economic development, representing the southern and northern regions of China. Then, A developed city and an underdeveloped city were selected from each province. Two hospitals and two primary healthcare institutions were randomly selected in each sample city. At last, 14 institutions have been included. Respondents are required to be over 18 years old and diagnosed as T2DM The guidelines proposed by Johnson and Orme (18) suggested that the sample size can be calculated using the equation: *N* > 500 × *c*/(*t* × *a*), where 500 is a constant, *c* represents the maximum number of levels in any attribute, *t* represents the count of DCE questions in each survey, and *a* signifies the number of choices in each DCE question. According the need of this study, we set *c* = 6, *t* = 6, and *a* = 2. Consequently, a theoretical sample size of no less than 250 have met the experimental requirements.

### Attributes and levels development

After conducting a literature review and examining *Clinical Guidelines for the Prevention and Treatment of Type 2 Diabetes in China (2022 Edition)*, we identify 10 attributes for diabetes treatment: treatment efficacy/reduction in HbA1c, hypoglycemic risk, mode of administration, medication frequency, out-of-pocket cost, Gastrointestinal adverse events, cardiovascular benefits, impaired liver and kidney function, weight change, and edema. Then, we conduct in-depth interviews with T2DM and organize a focus group discussion with 10 experts (senior: sub-senior: intermediate is 4:3:3) actively involved in clinical treatment of diabetes. Finally, seven attributes and their corresponding levels were included in this study (Table 1).

**Table 1:** Attributes and levels in the discrete-choice experiment survey.

Attributes	Levels	Description
Treatment efficacy/reduction in HbA1c	Poor (0.5%)	Different diabetes drugs have different efficacies for reducing the HbAIc.
	Intermediate (1%)	
	High (1.5%)	
	Highest (2.5%)	
Hypoglycemic risk	None (0%)	The likelihood that patients will experience mild or moderate hypoglycemic events within the first 6 months of use.
	Low (5%)	
	Medium (15%)	
	Higher (30%)	
Gastrointestinal adverse events	None (0%)	The likelihood that the medication will causes any mild or moderate Gastrointestinal adverse events (which may include diarrhea, vomiting and/or nausea) within the first 6 months of use.
	Low (10%)	
	Medium (20%)	
	Higher (40%)	
Weight change	−2 kg	Medication-related weight changes that patients will experience within the first 6 months of use.
	0 kg	
	+1.5 kg	
	+3 kg	
Cardiovascular benefits	No	In the first 6 months, the possible cardiovascular effects of drugs (including respiratory failure and thermoregulation).
	Yes	
Mode of administration	Injection	Provide pill and injection medication.
	Pill	
Out-of-pocket cost^*^	¥ 0	Patients’ monthly out-of-pocket cost.
	¥ 50	
	¥ 100	
	¥ 200	
	¥ 400	
	¥ 600	

* Based on a currency exchange rate of ¥ 7.02 to US$ 1.00 in 2022.

### Experimental design

DCE as a way to quantify preferences is increasingly advocated for health field (7). Patients are asked to select their preferred (and/or least preferred) alternative from a set of alternatives. DCEs are grounded in theories which assume that: Each option can be explained by several attributes; The preference value of the research object depends on the level of these attributes; The respondents made their choices based on the utility maximization. The utility value that respondents derive from a medication plan can be represented by the following equation:


$$U_{ji}=\beta_{0}+X_{1ji}+\beta_{2}X_{2ji}+\beta_{m}X_{mji}+\varepsilon_{ji}$$

where $U_{ji}$ is a function of *i* diabetes treatment-related attributes ( $X_{1ji}$ … $X_{mji}$ ), and $\varepsilon_{ji}$ is the residual term. We obtain the coefficient values ( $\beta_{0}\ldots \beta_{m}$ , $\beta_{0}$ constant terms) for the above equations through a statistical analysis model. These coefficients indicate the change in marginal utility when different attribute levels change. The choice sets were constructed using a D-efficient design to maximize statistical efficiency and minimize the number of required choices in SAS 9.4. Attribute levels were determined based on a comprehensive literature review and consultation with clinical experts to ensure they were relevant and representative of real-world scenarios. To ensure data quality, we establish a set of DCE questions with “clear advantages (dominant DCE task),” which all levels of Drug 1 being superior to Drug 2. If Drug 2 was selected, it is considered invalid (Table 2).

**Table 2:** Example of DCE selection set.

Attributes/levels	Drug A	Drug B
Treatment efficacy/reduction in HbA1c	Intermediate (1%)	Poor (0.5%)
Hypoglycemic risk	Low (5%)	Medium (15%)
Gastrointestinal adverse events	Medium (20%)	Low (10%)
Weight change	0 kg	+3 kg
Cardiovascular benefits	Yes	No
Mode of administration	Injection	Pill
Out-of-pocket cost	¥50	¥ 100
Which of the two drugs on the right do you prefer?	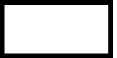	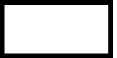

### Data and statistical analysis

To guarantee the data quality, we conduct a preliminary examination involving 30 T2DM patients before the study and conduct face-to-face on-site questionnaire surveys for T2DM collaborating with physicians. A total of 1,443 questionnaires are collected in this study, 34 questionnaires do not pass the DCE consistency test. The effective questionnaire rate is 97.6%. The research adheres to the ethical principles outlined in the Helsinki Declaration and has received approval from the Ethics Review Committee of the School of Public Health at Fudan University. Each respondent provided an informed consent form before being included in the study.

Epidata 3.1 and Stata 16.0 were used for data organization and statistical analysis. Two econometric approaches were used to estimate this utility function, including the classical conditional logit model (CLM) and a mixed logit model (MIXL) that could be used to capture potential unobservable preference heterogeneity. The optimal model was chosen by examining the Bayesian information criterion (BIC) and Akaike information criterion (AIC). The results indicate that the mixed logit model offers a superior fit for the existing data. The values of AIC and BIC are 4,986.529 and 5,119.758, respectively. The signs of coefficients of the model indicated whether the corresponding attributes had a positive or negative effect on utility, and the sizes of the coefficients indicated the attributes relative importance based on total relative utility. The cost attribute was assumed to be continuous (19). Thus, the marginal willingness to pay (WTP) was calculated by assessing the ratio of the preference for other attributes to the preference for out-of-pocket cost under UEBMI and URRBMI.

## Results

### Basic information of the respondents

In the UEBMI group, 437 participants (56.2%) were male, and 733 respondents (94.3%) resided in urban areas. The majority of respondents (51.5%) preferred pills as their method of medication, with 25.6% spending ¥100–¥200 per month on hypoglycemic drugs.

In the URRBMI group, 290 participants (45.9%) were male, and 269 respondents (42.6%) resided in urban areas. Similarly, 52.7% preferred pills, with 26.3% spending ¥100–¥200 per month on hypoglycemic drugs (Table 3).

**Table 3:** Basic personal information of respondents.

	UEBMI (*N* = 777)		URRBMI (*N* = 632)	
	Frequency	Ratio (%)	Frequency	Ratio (%)
Gender				
Male	437	56.2	290	45.9
Female	340	43.8	342	4.1
Registered residence				
Cities and towns	733	94.3	269	42.6
Village	44	5.7	363	57.4
Mode of taking medicine				
Pill	399	51.4	333	52.7
Injection	85	10.9	73	11.6
Not taking medicine	78	10.0	61	9.7
Pill and injection	215	27.7	165	26.1
The monthly cost of purchasing hypoglycemic drugs				
¥ 0–¥ 50	64	8.2	46	7.3
¥ 50–1¥ 00	148	19.0	107	16.9
¥ 100–¥ 200	199	25.6	165	26.3
¥ 200–¥ 400	155	19.9	164	5.9
¥ 400–¥ 600	93	12.0	68	10.8
>¥ 600	62	8.0	38	6.0
Not purchasing	56	7.2	44	7.0

### Preference weight and WTP

**Figure 1:**
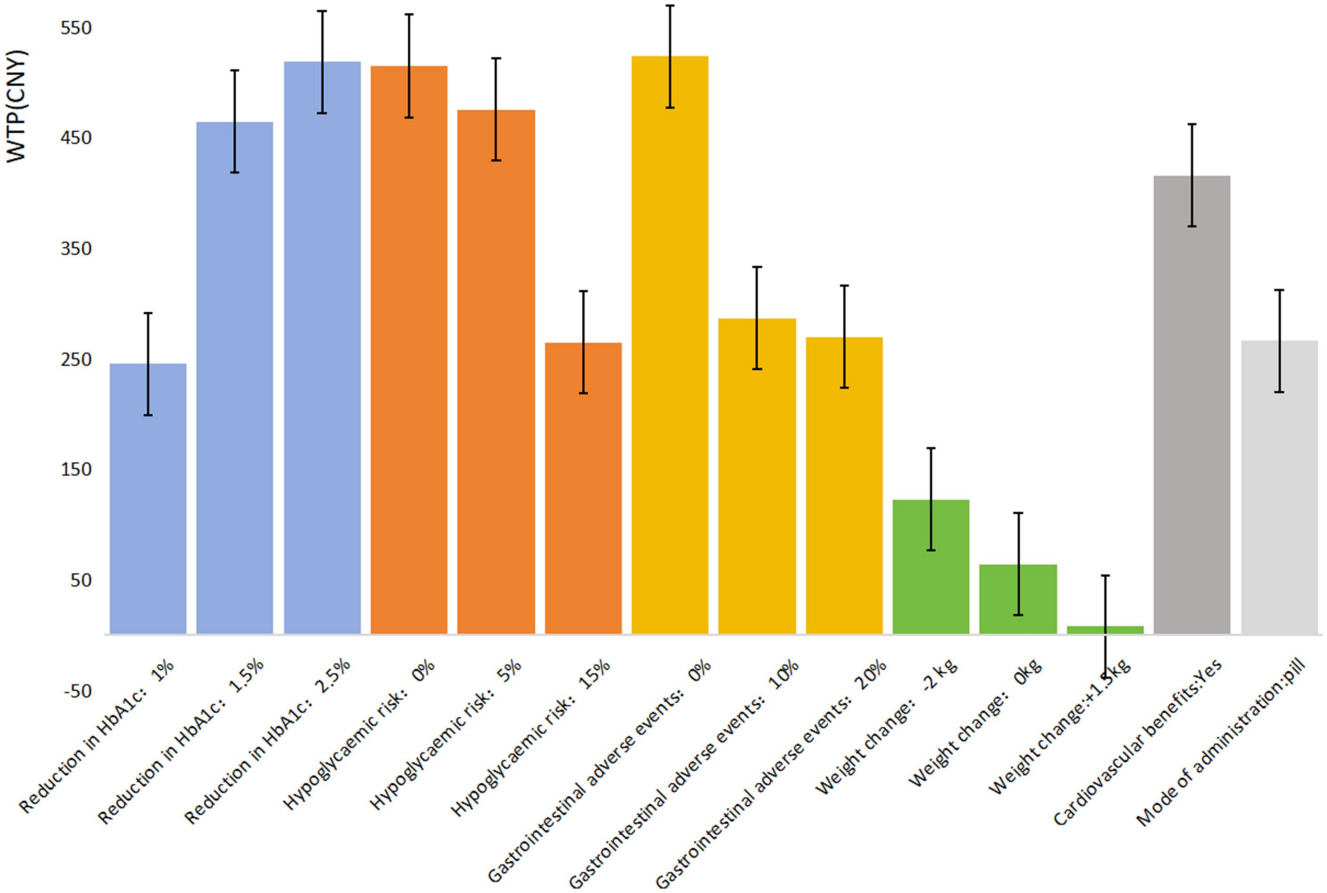
WTP of T2DM patients that are covered by UEBMI for each attribute/level.

The mixed logit model results (Table 4) show that all seven attributes were statistically significant (*p* < 0.05) for patients with UEBMI. The most critical attribute for these patients was gastrointestinal adverse events, followed by treatment efficacy/reduction in HbA1c, hypoglycemic risk, cardiovascular benefits, mode of administration, and weight change. Patients placed the highest importance on avoiding gastrointestinal adverse events, strongly preferring medications with a 0% risk of such events (*β* = 1.816, *p* < 0.001). If the risk of gastrointestinal adverse events is reduced from 40% to 0%, patients are willing to pay an additional ¥523.49 per month. Similarly, treatment efficacy was highly valued, with patients favoring a 2.5% reduction in glycated hemoglobin A1c (HbA1c) levels (*β* = 1.798, *p* < 0.001). When efficacy improves from a 0.5% to a 2.5% reduction in HbA1c, patients are willing to pay an additional ¥518.44 per month. Regarding hypoglycemic risk, patients showed a strong preference for medications with 0% risk of hypoglycemia (*β* = 1.785, *p* < 0.001), being willing to pay ¥514.71 more per month for such options. They also expressed a preference for medications offering cardiovascular protection (*β* = 1.441, *p* < 0.001), with a willingness to pay ¥415.48 per month for this benefit. In comparison, the mode of administration and weight change were less influential in patients’ decisions regarding hypoglycemic drug choice (Figure 1).

**Table 4:** Mixed logit model for respondent with UEBMI preferences.

Attributes/levels	Estimate	SE	*p*-value	SD	SE	*p*-value
Treatment efficacy/reduction in HbA1c: poor (0.5%) (ref.)						
Intermediate (1%)	0.851	0.163	<0.001	0.779	0.351	0.027
High (1.5%)	1.610	0.236	<0.001	0.485	0.511	0.342
Highest (2.5%)	1.798	0.250	<0.001	1.565	0.336	<0.001
Hypoglycemic risk: higher (30%) (ref.)						
None (0%)	1.785	0.246	<0.001	0.980	0.378	0.010
Low (5%)	1.648	0.237	<0.001	0.253	0.574	0.659
Medium (15%)	0.919	0.175	<0.001	0.046	0.322	0.885
Gastrointestinal adverse events: higher (40%) (ref.)						
None (0%)	1.816	0.267	<0.001	1.627	0.342	<0.001
Low (10%)	0.994	0.180	<0.001	0.203	0.402	0.613
Medium (20%)	0.935	0.174	<0.001	0.794	0.330	0.016
Weight change: +3 kg (ref.)						
-2 kg	0.425	0.141	<0.003	0.883	0.357	0.013
0 kg	0.222	0.160	<0.166	1.430	0.316	0.000
+1.5 kg	0.028	0.135	<0.838	0.210	0.284	0.457
Cardiovascular benefits: no (ref.)						
Yes	1.441	0.196	<0.001	1.697	0.236	<0.001
Mode of administration: injection (ref.)						
Pill	0.923	0.143	<0.001	1.708	0.257	<0.001
Out-of-pocket cost	−0.0034	0.0005	<0.001	0.005	0.001	<0.001
Observed value	9,024					
AIC/BIC	AIC: 4,986.529 BIC: 5,119.758					
Wald chi^2^	284.63					
Log likelihood	−2,423.2644					

Table 5 shows that all seven attributes significantly influence the medication preferences of T2DM patients covered by URRBMI (*p* < 0.05). The most important attribute for URRBMI patients is treatment efficacy/reduction in HbA1c, followed by gastrointestinal adverse events, hypoglycemic risk, cardiovascular benefits, mode of administration, and weight change. URRBMI patients prioritize treatment efficacy, showing a strong preference for a 2.5% reduction in glycated hemoglobin A1c (HbA1c) (*β* = 1.603, *p* < 0.001). They are willing to pay ¥328.33 more per month for medications with the highest efficacy (2.5% reduction in HbA1c) compared to the lowest (0.5% reduction).

**Table 5:** Mixed logit model for respondent with URRBMI preferences.

Attributes/levels	Estimate	SE	*p*-value	SD	SE	*p*-value
Treatment efficacy/reduction in HbA1c: poor (0.5%) (ref.)						
Intermediate (1%)	0.808	0.133	<0.001	0.486	0.335	0.147
High (1.5%)	1.366	0.154	<0.001	0.338	0.512	0.508
Highest (2.5%)	1.603	0.173	<0.001	1.077	0.222	<0.001
Hypoglycemic risk: higher (30%) (ref.)						
None (0%)	1.119	0.148	<0.001	0.062	0.270	0.818
Low (5%)	0.961	0.139	<0.001	0.046	0.305	0.880
Medium (15%)	0.648	0.131	<0.001	0.082	0.289	0.778
Gastrointestinal adverse events: higher (40%) (ref.)						
None (0%)	1.302	0.169	<0.001	1.348	0.235	<0.001
Low (10%)	0.895	0.145	<0.001	0.190	0.391	0.627
Medium (20%)	0.384	0.121	0.001	0.243	0.641	0.705
Weight change: +3 kg (ref.)						
−2 kg	0.395	0.117	<0.001	0.005	0.298	0.985
0 kg	0.361	0.129	<0.005	0.048	0.515	0.926
+1.5 kg	0.236	0.122	<0.052	0.326	0.325	0.317
Cardiovascular benefits: no (ref.)						
Yes	0.916	0.105	<0.001	1.150	0.148	<0.001
Mode of administration: injection (ref.)						
Pill	0.588	0.093	<0.001	1.180	0.150	<0.001
Out-of-pocket cost	−0.004	0.001	<0.001	0.005	0.001	<0.001
Observed value	7,404					
AIC/BIC	AIC: 3,924.46 BIC: 4,131.75					
Wald chi^2^	285.48					
Log likelihood	−1,932.2288					

**Figure 2:**
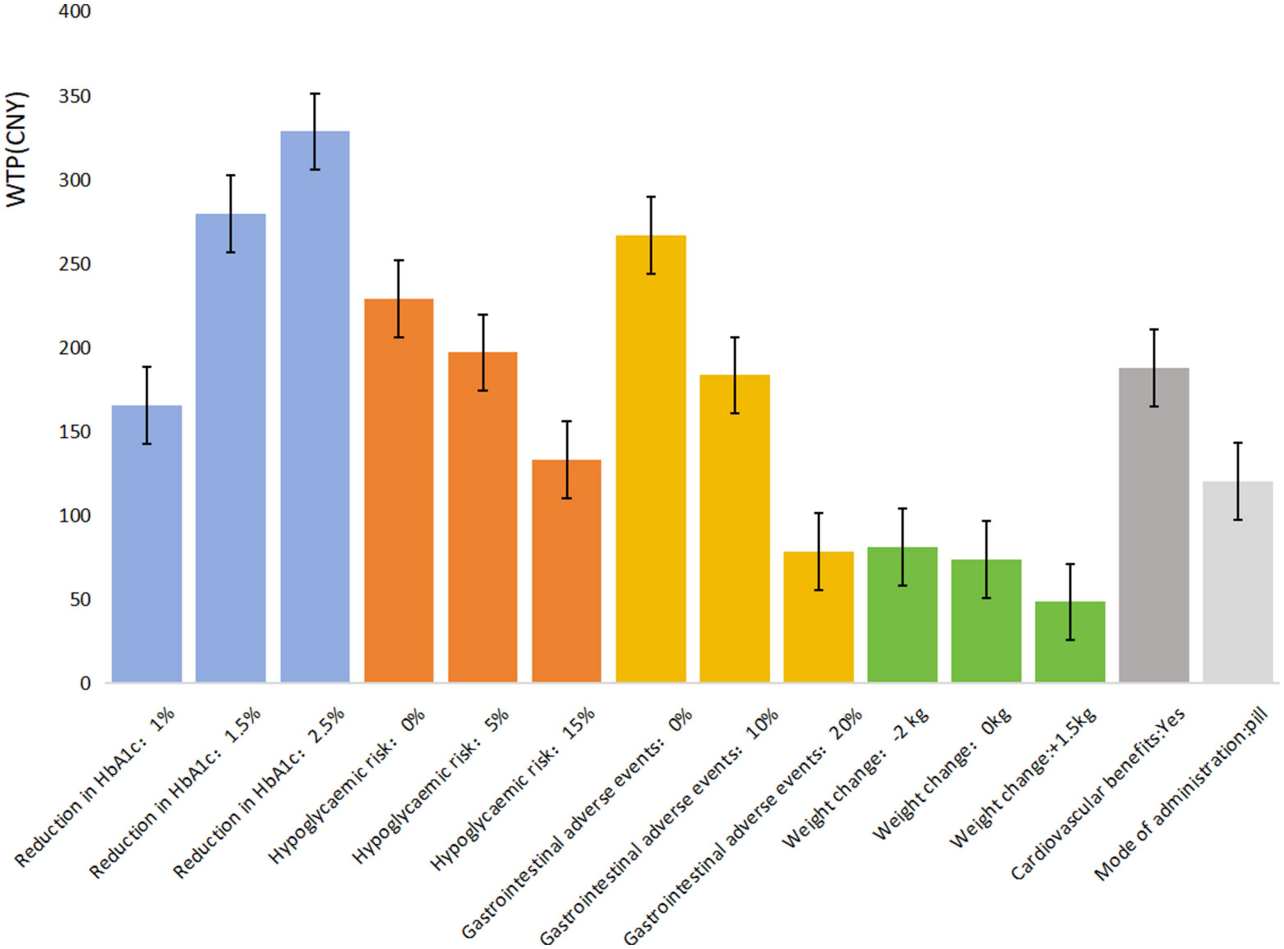
WTP of T2DM patients that are covered by URRBMI for each attribute/level.

Additionally, they prefer medications that do not cause gastrointestinal adverse events (0%) (*β* = 1.302, *p* < 0.001), with a willingness to pay an extra ¥266.62 when the risk is reduced from 40% to 0%. Regarding hypoglycemic risk and cardiovascular benefits, URRBMI patients favor medications with 0% hypoglycemic risk (*β* = 1.119, *p* < 0.001) and those that provide cardiovascular protection (*β* = 0.916, *p* < 0.001), expressing a willingness to pay ¥514.71 and ¥187.45, respectively, for these benefits. The mode of administration and weight change are the least influential factors in their medication choices (Figure 2).

In comparing patients covered by UEBMI and URRBMI, notable differences emerge in their preferences for diabetes medications. UEBMI patients place the highest emphasis on avoiding gastrointestinal adverse events, with a willingness to pay ¥523.49 to reduce the risk to 0%, which is ¥5.05 higher than their willingness to pay for the highest level of treatment efficacy (2.5% reduction in HbA1c). In contrast, URRBMI patients prioritize treatment efficacy, with a maximum willingness to pay of ¥328.33 for a 2.5% HbA1c reduction, which is ¥99.33 higher than their willingness to pay to avoid gastrointestinal adverse events.

The willingness to pay (WTP) for improved medication quality also varies between these groups. For highest treatment efficacy (2.5% HbA1c reduction), UEBMI patients are willing to pay ¥190.11 more than URRBMI patients. Conversely, UEBMI patients are willing to pay ¥256.87 more than URRBMI patients to avoid gastrointestinal adverse events. Overall, UEBMI patients tend to be willing to pay more than URRBMI patients for medications with better safety and efficacy profiles.

## Discussion

Effective communication between healthcare providers and patients is essential for optimal diabetes management. Understanding the different medical insurance coverages and medication preferences of T2DM patients facilitates shared decision-making, ensuring that treatment choices align with patient needs and improve adherence. This study employed DCE methodology to examine the medication preferences of T2DM patients with UEBMI and URRBMI, revealing distinct tendencies linked to their insurance types. Our findings indicate that gastrointestinal adverse events are the primary concern for UEBMI patients, whereas URRBMI patients prioritize treatment efficacy.

Hypoglycemic risk also significantly affects medication preferences, ranking third in importance for both insurance groups. The risk of severe hypoglycemia poses challenges to daily living and can have life-threatening consequences (20). Our study suggests that UEBMI patients are willing to pay more for reducing this risk, indicating a preference for medications that balance effectiveness with safety. When developing treatment plans, healthcare professionals should prioritize drugs that minimize gastrointestinal side effects and hypoglycemic risks, thus enhancing overall patient satisfaction.

Furthermore, patients with UEBMI express a greater preference for medications that offer cardiovascular protection, which is crucial given the heightened risk of heart failure associated with T2DM (21). The combination of diabetes and cardiovascular disease leads to poorer health outcomes and increased mortality rates (22). Therefore, it is essential to consider cardiovascular protection in medication choices, particularly for patients with UEBMI. Based on our findings, we recommend policy changes aimed at expanding access to preferred medications, especially for patients covered under different insurance schemes. Policymakers should consider revising reimbursement policies and coverage limitations to ensure that patients have equitable access to medications that align with their preferences and clinical needs.

In terms of mode of administration, oral medications are preferred, enhancing adherence by allowing patients to take their medications conveniently (23). Our results indicate that patients with URRBMI show a higher willingness to pay for oral medications rather than injections, emphasizing the importance of convenience in treatment choices. Policymakers could adopt a more patient-centered approach by considering both cost-effectiveness and patient preferences to create more balanced insurance schemes that enhance patient satisfaction and treatment adherence.

Lastly, while changes in weight during the first 6 months of treatment have limited influence on medication preferences, maintaining a healthy weight is critical for effective diabetes management (24). Healthcare providers should engage in discussions about weight management alongside medication efficacy, ensuring that patients are informed about the implications of weight changes on their health.

For health insurance policymakers, the results suggest the need for more tailored insurance schemes that consider the diverse preferences and priorities of different patient groups, such as UEBMI and URRBMI patients, to enhance patient-centered care and improve treatment adherence. For clinicians, the findings highlight the importance of considering patient preferences, especially regarding side effects and treatment effectiveness, during shared decision-making. By aligning treatment recommendations with patient preferences, clinicians can potentially improve patient satisfaction and clinical outcomes.

Despite implementing strict quality control measures before the on-site investigation and data analysis, this study has several limitations. First, the geographic scope may restrict the generalizability of the findings across different regions in China, particularly between urban and rural areas, as respondents are drawn from only two provinces. Future studies could explore the generalizability of our findings by including a broader range of patient populations and health conditions. Additionally, confounding factors, such as traditional beliefs and cultural influences, were not sufficiently addressed, leaving open the question of how these factors might independently affect medication preferences beyond insurance coverage. Potential biases in self-reported data may also compromise the accuracy of the stated preferences.

## Conclusion

This research employs DCE to assess medication preferences among patients with T2DM covered by UEBMI and URRBMI. The results reveal that the most significant factors influencing T2DM patients are gastrointestinal adverse events and treatment efficacy, followed closely by the risk of hypoglycemia. Additionally, medication preferences differ considerably between patients with these two types of insurance. These insights are crucial for enhancing medication adherence among T2DM patients and will assist healthcare professionals in making informed decisions regarding drug therapies.

## Data Availability

The original contributions presented in the study are included in the article/supplementary material, further inquiries can be directed to the corresponding authors.
